# A study on the clinical outcomes of patients with revision surgery for adjacent segment disease after 10-year’s anterior cervical spine surgery

**DOI:** 10.1186/s13018-016-0341-x

**Published:** 2016-01-13

**Authors:** Jia Li, Tong Tong, Ruijie Niu, Yong Shen

**Affiliations:** Department of Spine Surgery, The Third Hospital of Hebei Medical University, Shijiazhuang, 050051 People’s Republic of China; The Key Laboratory of Orthopedic Biomechanics of Hebei Province, The Third Hospital of Hebei Medical University, Shijiazhuang, 050051 People’s Republic of China

**Keywords:** Anterior cervical discectomy and fusion, Anterior cervical corpectomy and fusion, Adjacent segment disease, Reoperation, Outcomes

## Abstract

**Background:**

The purpose of this study was to investigate the clinical outcomes of anterior cervical discectomy and fusion (ACDF) or anterior cervical corpectomy and fusion (ACCF) as a revision surgery for adjacent segment disease (ASD) after primary surgery.

**Methods:**

There were 35 patients who underwent anterior cervical spine surgery for symptomatic recurrent radicular or myelopathic symptoms from ASD. According to the ASD involved levels superior or inferior to the previous operated level, patients were divided into two groups: superior and inferior groups. The patients were also grouped into ACDF and ACCF groups by who received ACDF or ACCF as revision surgery for ASD. Clinical evaluations were performed preoperatively and repeated at 2 years after operation.

**Results:**

In this study, a total of 35 patients with a minimum of 2 years of follow-up data were available for analysis. There were 20 patients in the superior group and 15 patients in the inferior group according to the ASD developed at levels. Of these 35 patients, according to the treatment method, 12 patients were in the ACCF group and 23 patients were in the ACDF group. The Japanese Orthopaedic Association (JOA), Neck Disability Index (NDI), and visual analogue scale (VAS) on arm pain and neck pain scores demonstrated significant improvement compared to the preoperative scores in both groups (superior and inferior groups or ACDF and ACCF groups) (*P* < 0.05). However, there was no difference between the two groups (superior and inferior groups or ACDF and ACCF groups) (*P* > 0.05).

**Conclusions:**

According to our study, both superior and inferior adjacent-level groups together with ACDF and ACCF groups maintained favorable clinical results on patients who underwent one-level ACDF for symptomatic new radicular or myelopathic symptoms.

## Background

Anterior cervical discectomy and fusion (ACDF) and anterior cervical corpectomy and fusion (ACCF) have been used widely as an anterior approach for the surgical treatment of cervical spondylotic myelopathy (CSM) in the past years [1–3]. ACDF could remove the herniated disc tissue and decompress the anterior spinal cord, which is associated with a low prevalence of graft extrusion or migration. When the spinal cord compression is located behind the vertebral body, especially large posterior osteophyte adjacent to the endplate, ACCF performed as an alternative technique. It could achieve better access and more extensive decompression, which provide more bony autograft to promote fusion [4–6]. Biomechanical and clinical studies suggested that adjacent-level kinematic might predispose to adjacent-level degeneration after ACDF [7, 8]. Degenerative changes in adjacent-level and unsatisfactory clinical outcomes were documented [9, 10]. Hilibrand et al. defined adjacent segment disease (ASD) as correlation between the development of new radiculopathy or myelopathy and new imaging evidence of degenerative changes at levels adjacent to the previous arthrodesis of the cervical spine [11, 12]. Lawrence et al. concluded that the risk of developing new symptoms secondary to adjacent segment pathology causing radiculopathy and/or myelopathy after cervical fusion surgery ranged from a cumulative rate of 1.6 to 4.2 % per year [13]. Goffin et al. reported that in the basis of more than 60 months follow-up, 92 % of the patients who were treated by ACDF demonstrated degenerative changes at the adjacent levels [14]. In 2009, Matsumoto et al. performed a prospective MRI study, involving in patients who underwent ACDF and healthy control subjects at 10-year follow-up. During 10 years, both ACDF patients and control subjects demonstrated progression of disc degeneration. ACDF patients had significantly higher incidence of progression of disc degeneration at adjacent segments than control subjects, while progression of disc degeneration at adjacent segments was not always related to development of clinical symptoms [15]. However, few studies had compared the use of the two decompressive techniques for the treatment of ASD after anterior cervical spine surgery. Therefore, the purpose of this study was to investigate the clinical outcomes of ACDF or ACCF as a revision surgery for ASD after primary surgery.

## Methods

From January 1990 to December 2006, a total of 1132 patients underwent ACDF for cervical degenerative diseases in the authors’ institution. Thirty-five patients underwent revision ACDF or ACCF for ASD between January 2005 and December 2011. All the patients had no expression of already existing degeneration (radiographic evidence of degenerative changes; radicular or myelopathic signs and symptoms that correlate with imaging evidence of degeneration) at the time of the first surgery. This study had been approved by Ethics Committee of The Third Hospital of HeBei Medical University, and all patients signed informed consent.

Treatment of patients with ASD followed the same principles as those of patients with primary cervical spondylosis. These patients who developed gradual neurological changes followed 6 months of invalid conservative treatment (such as physical therapy and drugs). The selection for ACDF or ACCF was determined by the presence or absence of retrovertebral compression. If it was easier to remove the compression using a corpectomy, then the ACCF was performed. Otherwise, the ACDF was selected. Patients with cervical spine trauma, tumor spinal pathologies, neoplasm, spinal infections, congenital deformations, and chronic systemic illnesses such as rheumatoid arthritis and neurodegenerative diseases were excluded in this study. Patients with ASD (multisegmental spinal cord compression or ossification of the posterior longitudinal ligament) that needed to be decompressed using a posterior approach were also excluded.

### Surgical technique

All patients received ACDF by the same senior surgeon. Revision surgical procedures were carried out using the anterior approach via a right-sided skin incision, provided that the adjacent segments of primary surgery level needed reoperation. Due to serious neural decompression, resection of the osteophytes, posterior longitudinal ligament, and disc must be excised completely. The endplates were resected with a curette or burr. In some cases, dural ossification and adhesion of the dura to the ligament made the separation more difficult. In these cases, the “floating method” was the approach of choice. The polyetheretherketone (PEEK) cage or titanium mesh cage (Medtronic Sofamor Danek, Memphis, TN) was used, which was filled with local bone fragments from the decompression and inserted into the disc space and the anterior plate system was applied (Medtronic Sofamor Danek, Memphis, TN).

### Evaluation criteria

**Fig. 1:**
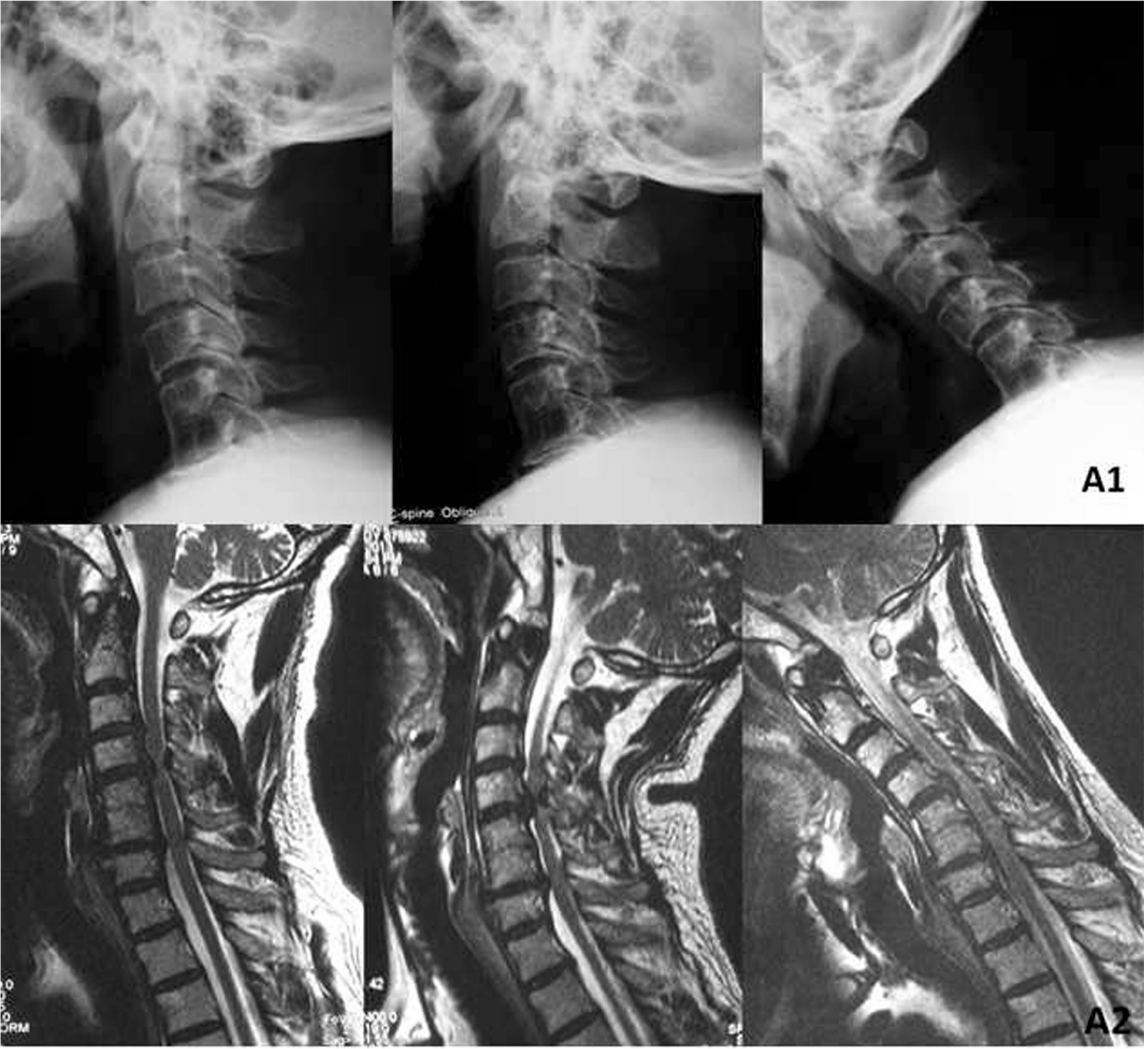
A 67-year-old man developed one-level adjacent segment disease 11 years after primary surgery. (*A1*) Radiograph and (*A2*) MRI (flexion/extension) at 11 years after operation indicates C3-4, C4-5 disc hernias

**Fig. 2:**
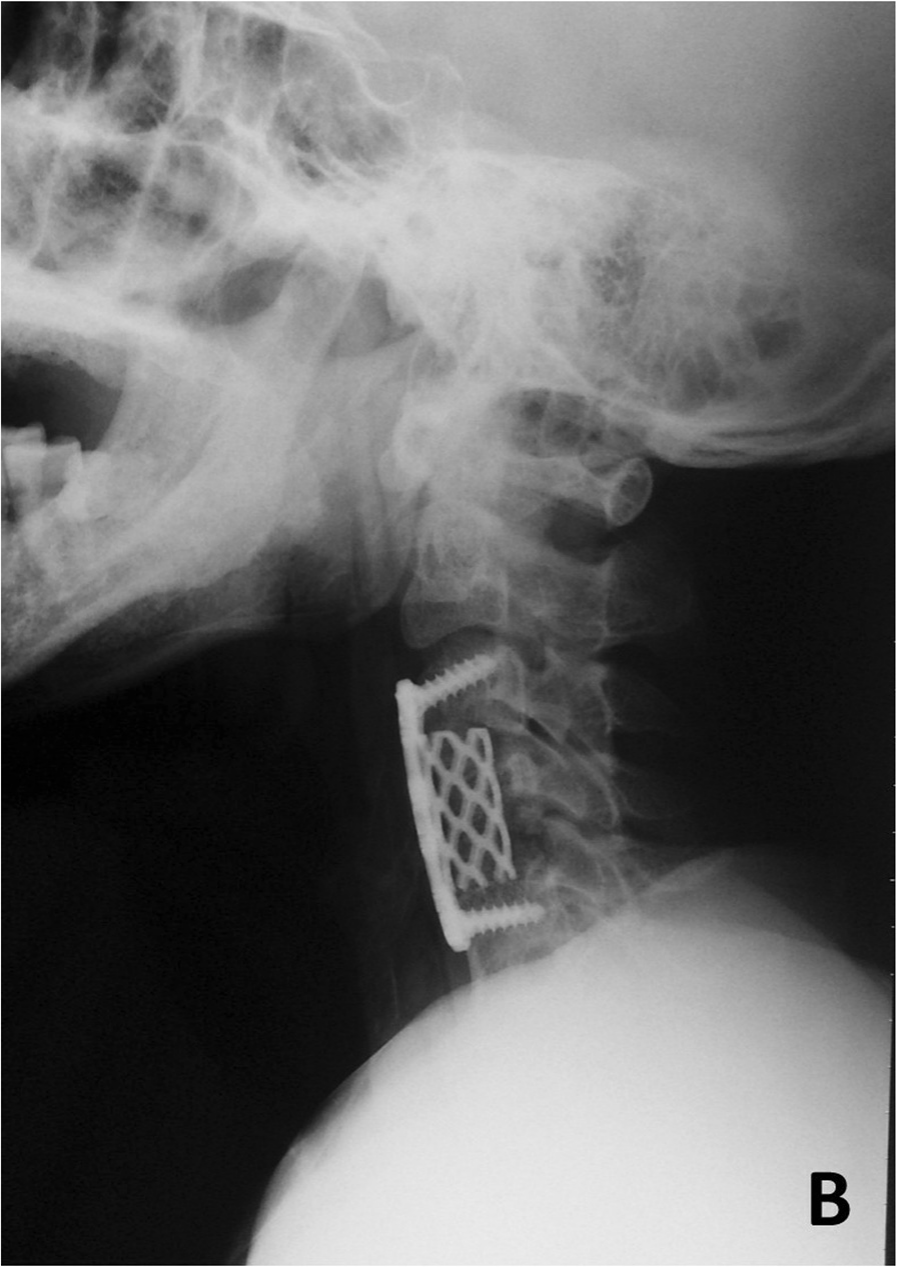
*B* Anterior cervical interbody fusion was performed again

**Fig. 3:**
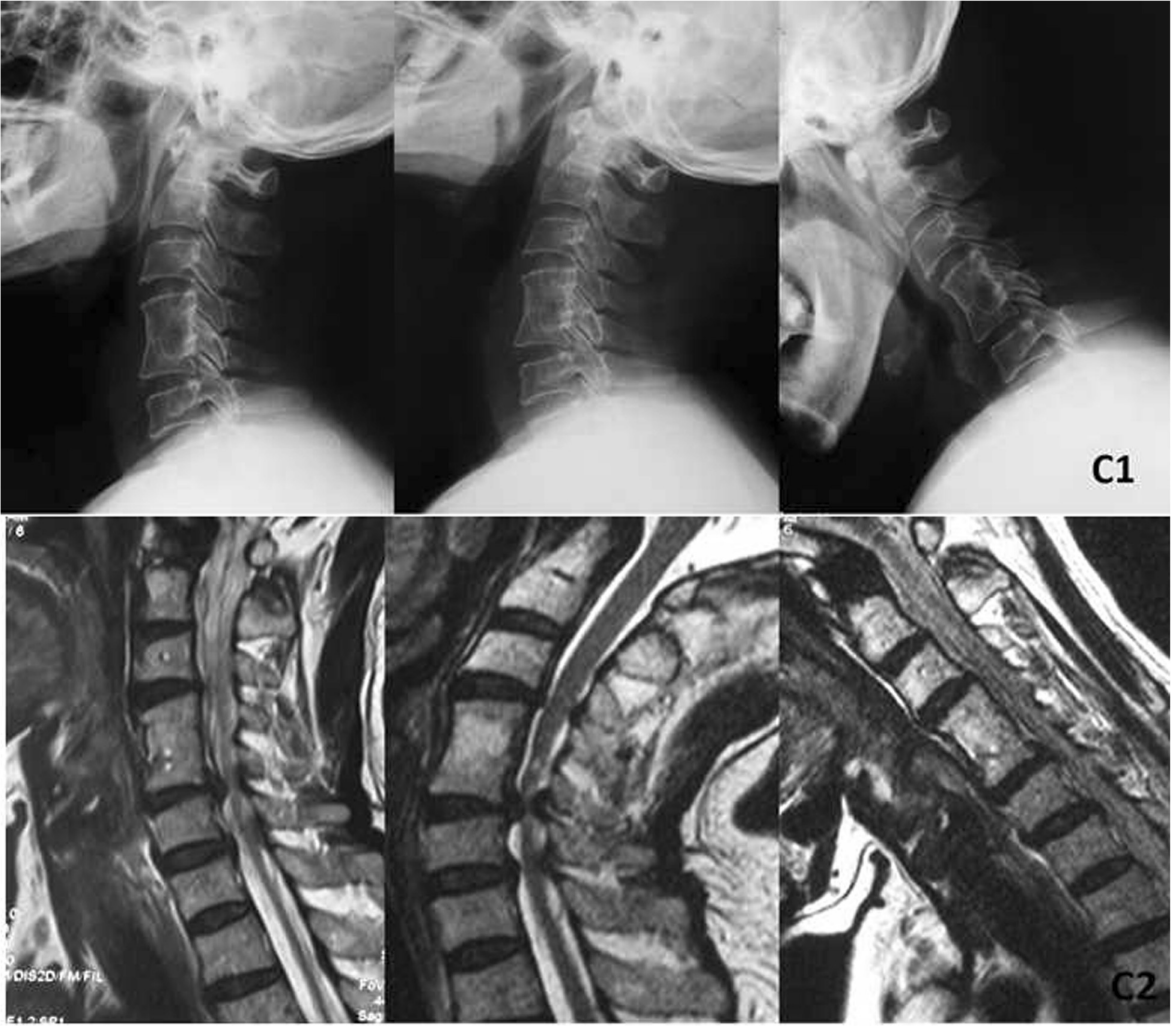
A 58-year-old woman developed one-level adjacent segment disease 12 years after primary surgery. (*C1*) Radiograph and (*C2*) MRI (flexion/extension) at 12 years after operation indicate C5–6 disc hernias

**Fig. 4:**
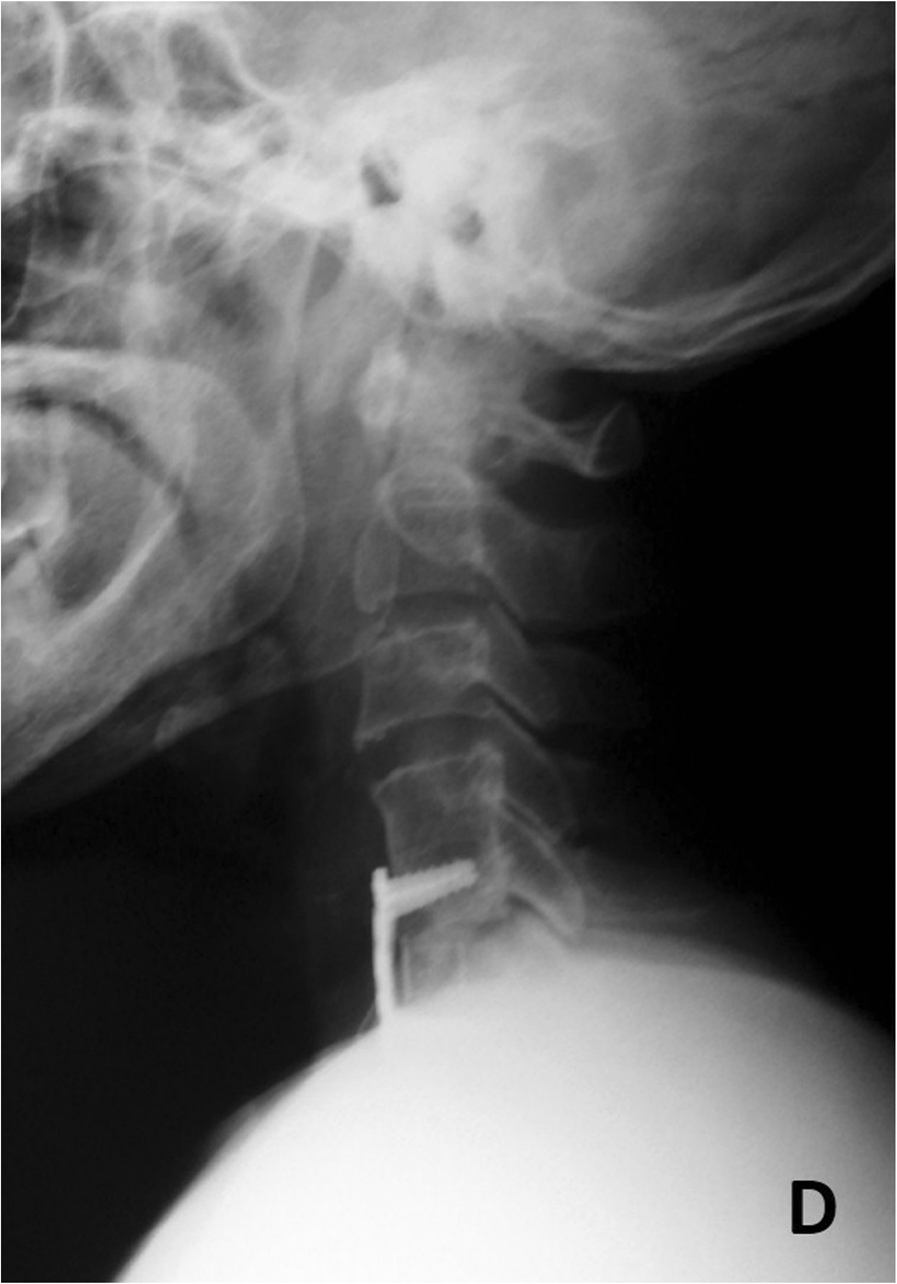
*D* Anterior cervical interbody fusion was performed again

Clinical data including clinical and radiological evaluation results were collected preoperatively and at 3, 6, 12, and 24 months after surgery. When the follow-up was longer than 2 years, the last data available were used for statistical analysis. Radiographic evaluation included static and dynamic flexion/extension lateral images which were used to assess ASD by two independent doctors who evaluated the radiographs without knowledge of the clinical outcomes. ASD was defined as the following: calcification of the anterior longitudinal ligament, a narrowing of the disc space with or without posterior osteophytes, and new anterior or enlarging osteophyte formation [14]. The authors divided patients with ASD developed at levels superior or inferior relative to the index of ACDF level into two groups: superior and inferior groups. The authors divided patients with ASD who received ACDF or ACCF into two groups: ACDF and ACCF groups (Figs. 1, 2, 3, and 4).

The modified Japanese Orthopaedic Association (JOA) scoring system was used to determine functional status before surgery and at the final follow-up visit. The recovery rate (%) at the final follow-up visit was calculated by using the Hirabayashi method: (postoperative JOA score − preoperative score) / (17 − preoperative score) × 100 %. The normal score of JOA presents 17. Neck Disability Index (NDI) was used to understand how much the neck pain affected the ability to manage daily life. Visual analogue scale (VAS) was used to determine neck and arm pain before surgery and at the final follow-up visit.

### Statistical analysis

All data were collected, and the software of by SPSS Version 17.0 (SPSS Inc, Chicago, IL) was used for the statistical evaluation. A paired *t* test was used to identify a significant difference between pre- and postoperative measurements of JOA, NDI, and VAS for neck and arm pain for each group. The independent two-sample *t* test was used to identify a significant difference between the groups. In all analyses, significance was defined as *P* < 0.05. Results were presented as mean ± standard deviation.

## Results

This retrospective study has a total of 19 males and 16 females with a mean age at revision operation of 55.2 ± 6.8 years (range, 50–70 years). The patients were followed up with an average of 13.2 years (range 10–15 years). Before revision surgery, adjacent segment degeneration has been found in one segment in 20 patients and in two segments in 15 patients. There were 20 patients in the superior group and 15 patients in the inferior group. Of these 35 patients, 12 patients were in the ACCF group and 23 patients were in the ACDF group. No significant differences existed in age, sex, symptom duration, ASD level, or follow-up between either superior and inferior groups or the ACCF and ACDF groups (Tables 1 and 2). There were no cases of intraoperative complications, major neurological or vascular complications, and pseudoarthrosis or wound complications. The incidence of the dysphagia in patients was 2.8 %, and one patient with ASD reported mild dysphagia symptoms. No patient needed additional cervical decompressive surgery due to recurrent or residual symptoms. The surgical outcome was excellent in 9 (25.7 %) patients, good in 15 (42.9 %), fair in 8 (22.9 %), and poor in 3 (8.6 %). The rate of excellent and good outcomes reached 68.6 %. The JOA, NDI, and VAS on arm pain and neck pain scores demonstrated significant improvement compared to the preoperative scores in both superior and inferior groups (*P* < 0.05). However, there was no difference between the two groups (Table 3). The recovery rate was 67.9 and 66.3 % in patients who underwent superior and inferior procedures, respectively. There was no difference in preoperative JOA, NDI, and VAS on arm pain and neck pain between the ACDF and ACCF groups. Both groups reported significant improvements in JOA, NDI, and VAS on arm pain and neck pain from the preoperative means. But no differences were found between the groups (Table 4). The recovery rate was 68.2 and 66.7 % in patients who underwent ACDF and ACCF, respectively.

**Table 1:** Patient demographics (superior and inferior group)

Demographics	Superior group	Inferior group	*P* value
Age (year)	53.6 ± 5.7	56.5 ± 7.8	0.861
Sex			0.922
Men	11	8	
Women	9	7	
Primary fused level			0.124
C4–5(7)	7	6	
C5–6(18)	15	12	
C6–7(10)	9	1	
Duration of symptoms (months)	15.3 ± 3.8	16.9 ± 6.1	0.532
Follow-up (months)	25.6 ± 5.2	27.7 ± 3.8	0.317

**Table 2:** Patient demographics (ACDF and ACCF groups)

Demographics	ACDF group	ACCF group	*P* value
Age (year)	54.3 ± 6.3	56.1 ± 7.6	0.763
Sex			0.713
Men	13	6	
Women	10	6	
ASD levels			0.724
C3–4	4	3	
C4–5	8	7	
C5–6	6	9	
C6–7	7	5	
C7–T1	1	0	
Duration of symptoms (months)	15.9 ± 4.1	16.6 ± 5.8	0.285
Follow-up (months)	25.9 ± 5.6	26.9 ± 4.1	0.739

**Table 3:** Comparison of surgical results between superior and inferior fused group in patients with adjacent segment disease

Outcomes	Superior group		Inferior group	
	Preoperative	Last follow-up	Preoperative	Last follow-up
JOA	8.6 ± 2.4	14.3 ± 3.8^†^	8.4 ± 3.1	14.1 ± 3.8^†^
NDI	50.2 ± 8.6	16.9 ± 5.2^†^	49.3 ± 5.3	16.3 ± 4.6^†^
VAS for neck pain	6.7 ± 1.6	2.9 ± 1.5^†^	6.8 ± 3.3	2.3 ± 1.3^†^
VAS for arm pain	6.1 ± 3.2	1.9 ± 1.6^†^	6.3 ± 1.9	1.6 ± 1.5^†^

^†^Significant difference between baseline and 24 months after surgery using paired *t* test; *P* < 0.05

^*^Significant difference between Superior and Inferior groups using independent two-sample *t* test; *P* < 0.05

**Table 4:** Comparison of surgical results between ACDF and ACCF groups in patients with adjacent segment disease

Outcomes	ACDF group		ACCF group	
	Preoperative	Last follow-up	Preoperative	Last follow-up
JOA	8.5 ± 3.1	14.3 ± 3.3^†^	8.6 ± 3.1	14.2 ± 2.8^†^
NDI	51.3 ± 9.3	16.6 ± 4.9^†^	48.3 ± 6.3	15.5 ± 5.7^†^
VAS for neck pain	6.9 ± 2.1	2.6 ± 1.8^†^	6.8 ± 2.3	2.5 ± 1.8^†^
VAS for arm pain	6.3 ± 2.8	2.1 ± 1.3^†^	6.7 ± 2.1	2.2 ± 1.9^†^

^†^Significant difference between baseline and 24 months after surgery using paired *t* test; *P* < 0.05

^*^Significant difference between discectomy and corpectomy groups using independent two-sample *t* test; *P* < 0.05

## Discussion

In recent years, symptomatic adjacent-level disease after ACDF or ACCF had become a common challenge for surgeons. However, arthrodesis of index level which irreversibly destroyed the intervertebral disc after ACDF or ACCF resulted in the biomechanical changes of superior or inferior level of the spine [15–17]. Many biomechanical studies have shown that loss of mobility at index level resulted in the increase of the intradiscal pressure and range of motion (ROM) at superior or inferior adjacent segments [18, 19]. These studies have been correlated with clinical issues, documenting the development of degenerative changes in adjacent spinal levels following ACDF [18, 20, 21].

Hilibrand et al. reported that the prevalence of symptomatic ASD occurred at a relatively constant incidence of 2.9 % per year during the 10 years after ACDF. In this study, ASD might be a result of the natural history of the cervical degenerative disease [11, 12]. Singh et al. reported that 1 of 48 patients who underwent single-level ACDF (2.1 %) received a reoperation within a 2-year follow-up [22]. King et al. investigated 12,338 patients who received cervical spine surgery and reported a reoperation rate of 2.5 % per year for ASD [23]. In this current long-term study, 35 (6.7 %) patients were involved to investigate the outcomes of revision surgery after primary ACDF. The incidence of revision surgery was similarly compared with previous long-term follow-up studies which was reported by Hilibrand et al. (7 %) and Ishihara et al. (6 %) [12, 23, 24].

Hilibrand et al. also reported that the C5/6 and C6/7 spinal levels were with higher risk of developing symptomatic ASD than others [12]. In other studies, it was suggested that these levels were more likely to develop ASD. It was well established that each intervertebral disc undertook different intradiscal pressures and ROM in the cervical spine [7, 15, 19]. Bydon et al. suggested that the specific spinal level might be a degenerative spinal disease development, but was not directly related to surgery. It suggested that the ASD was not directly due to the level of the index fusion itself [25]. The biomechanical study reported vertebral levels adjacent to C5/C6-simulated ACDF in cadaveric cervical spines which exerted increased intradiscal pressures and ROM at both superior and inferior adjacent levels during both flexion and extension [26]. During flexion and extension of the cervical spine, C4/5 level had higher intradiscal pressures and increased ROM compared to C6/C7 level. Superior vertebral levels experienced higher intradiscal pressures and increased ROM. It suggested that fusion might cause increased stress and strain on neighboring motion segments, which potentially contributed to accelerated degeneration. In this study, there were 20 patients in the superior group and 15 patients in the inferior group. No difference was found in preoperative JOA, NDI, and VAS on arm pain and neck pain between the two groups.

The clinical results after the first ACDF surgery were reported to be excellent. Sugawara et al. reported that there were 80 % of cases who demonstrated excellent results, so did the study of Faldini et al. (78 %) [27, 28]. To our knowledge, the outcomes of patients who received revision surgery were also reported excellently by Saarinen et al. (72 %) [29]. In their report, 72 % of patients were satisfied with the result, which was better compared with our finding of 68.6 % of satisfied patients after the reoperation. In the current study, a total of 35 patients who underwent one-level ACDF for symptomatic new radicular or myelopathic symptoms from ASD results showed that the clinical symptoms were significantly improved after a 2-year follow-up. However, there was no difference between the groups at the final follow-up. Furthermore, ACDF or ACCF as a revision surgery provided excellent results compared to primary surgery. Therefore, revision surgery does not adversely affect clinical results when performing ACDF in patients with ASD.

ACDF could remove the herniated disc tissue with a low incidence of graft migration. However, ACDF often had higher chance of incomplete decompression behind the vertebral body and risk of pseudarthrosis. Due to the risk of incomplete decompression, the ACCF could make a more extensive decompression during surgery and better access to remove the osteophytes behind the vertebral body. It also could provide a source for bony autograft to promote fusion. Due to the fewer possible points for ventral plate screw fixation, it might be associated with a higher incidence of complications, including graft migration or extrusion, dural tears, and blood loss. Owing to serious neural decompression, resection of the osteophytes, posterior longitudinal ligament, and disc must be excised completely. In some cases, adhesion of the dura to the ligament and/or dural ossification might result in separating difficultly. In these cases, the “floating method” was a choice. In case the dura was widely ossified or adhesive, the floating method was used to minimize the extent of surgical invasion and damage to the venous plexus, thus avoiding disturbance of the nervous tissues and decreasing acute dural inflation and cerebrospinal fluid leakage. Furthermore, sufficient decompression range and cutting the dura while keeping the arachnoid intact might also improve the clinical outcomes. Lau et al. found that both ACDF and ACCF had achieved similar clinical and radiological outcomes after a 6-year follow-up [30]. Song et al. also demonstrated that both two techniques provide satisfactory clinical outcomes and fusion rates for the treatment of CSM [31]. In our series, no difference in JOA, NDI, and VAS on arm pain and neck pain between the ACDF and ACCF groups at the last follow-up. The recovery rate was 68.2 and 66.7 % in patients who underwent ACDF and ACCF, respectively. But no differences were found between the groups.

### Limitation

Our study has some limitations. This study was only a retrospective study with a small sample size to explore the reality of adjacent-level disease after previous cervical spine fusions. In the future study, we can explore the correlation between superior and inferior adjacent levels and curvature on the cervical spine. Prospective multiple-center studies, long-term data, control group, and heterotopic bone formation are needed to confirm the result. Furthermore, cervical total disc replacement was not included in this study. The larger scale study should be performed to confirm the result and reported the outcome difference between different segments since the kinematics are different at different levels.

## Conclusions

According to our study, both superior and inferior adjacent-level groups together with ACDF and ACCF groups maintained favorable clinical results on patients who underwent one-level ACDF for symptomatic new radicular or myelopathic symptoms.
